# Associations between the gut microbiome and diet, body composition, and glycemic profiles: a cross-sectional *post-hoc* analysis of the Personal Diet Study

**DOI:** 10.3389/fnut.2026.1851439

**Published:** 2026-08-03

**Authors:** Chan Wang, Lauren Berube, Margaret Curran, Mary Lou Pompeii, Lu Hu, Souptik Barua, Huilin Li, David E. St-Jules, Antoinette Schoenthaler, Eran Segal, Michael Bergman, Collin J. Popp

**Affiliations:** 1 Division of Biostatistics, Department of Population Health, New York University Langone Health, New York, NY, United States; 2 Department of Epidemiology, Public Health Nutrition Program, NYU School of Global Public Health, New York, NY, United States; 3 Department of Population Health, Center for Healthful Behavior Change, Institute for Excellence in Health Equity, New York University Langone Health, New York, NY, United States; 4 Division of Precision Medicine, Department of Medicine, New York University Langone Health, New York, NY, United States; 5 Department of Nutrition, University of Nevada, Reno, Reno, NV, United States; 6 Department of Computer Science and Applied Mathematics, Weizmann Institute of Science, Rehovot, Israel; 7 Department of Medicine, Holman Division of Endocrinology, Diabetes and Metabolism, New York University Langone Health, New York, NY, United States

**Keywords:** glycemic variability, gut microbiome, personal diet, precision nutrition, type 2 diabetes

## Abstract

**Background:**

The gut microbiome is implicated in obesity and type 2 diabetes (T2D), but how diet, body composition, glycemic status, and self-efficacy factors relate to the microbiome in high-risk individuals is not well characterized. The purpose of this post-hoc analysis is to examine the relationship between the gut microbiome and obesity-related metabolic factors, including body composition, resting energy expenditure (REE), and glycemic variability (GV).

**Methods:**

Data for this *post-hoc* cross-sectional analysis were obtained from adults with prediabetes and obesity enrolled in The Personal Diet study, a 6-month behavioral weight loss study. Pre-intervention assessments (*n* = 95) included the collection of fecal microbiome profile, demographics, socioeconomic status, physical and metabolic characteristics [fat mass, fat free mass (FFM), continuous glucose monitoring–derived GV, REE], self-efficacy and dietary intake. Gut bacterial alpha diversity, beta diversity, and genus level abundances were associated with these host and lifestyle variables using multivariate regression, permutational multivariate analysis of variance, and correlation analyses.

**Results:**

Participants were a mean age of 58 years old, mostly female (75.8%), with a mean BMI of 34.6 kg/m^2^ and mean HbA1c of 5.7%. Higher FFM was associated with greater alpha diversity, whereas higher BMI was associated with lower diversity (*p* < 0.05). Dietary factors were the most consistent correlates of gut microbial beta diversity. At the genus level, associations were observed for sex, metformin use, BMI, protein intake, and REE. The *Prevotella/Bacteroides* ratio was positively associated with total energy, sugar, and carbohydrate intake (g/day) and negatively associated with monounsaturated fat intake (g/day). Glycemic measures and self-efficacy were not associated with any genera.

**Conclusion:**

In adults with prediabetes and obesity, the gut microbiome at baseline was most strongly associated with diet, with additional associations observed for body composition and selected host characteristics. These findings underscore diet as a key correlate of gut microbiome structure in a high-risk metabolic population and support further development of microbiome-informed precision nutrition strategies for obesity prevention and management.

## 1 Introduction

The microbiome refers to the collective genomes and ecological community of microorganisms—bacteria, archaea, viruses, and fungi—that live on and within the human body. The gut microbiome, residing primarily in the intestines, plays a central role in host metabolism, immune function, and overall health. Advances in high-throughput sequencing have revealed that inter-individual variation in microbiome composition and function is substantial and has been linked to a wide range of metabolic phenotypes, including obesity, insulin resistance, and type 2 diabetes (T2D) (1–4).

A key pathway connecting the gut microbiome to metabolic health is postprandial glycemic response (PPGR). Repeatedly elevated PPGRs can trigger downstream biological cascades—including increased insulin secretion, enhanced fat storage, and alterations in appetite regulation—that promote weight gain and contribute to the development of obesity and T2D (5). The gut microbiome has been shown to contribute extensively to interindividual differences in PPGR, even when individuals consume identical meals, suggesting that microbiome-informed approaches may improve prediction and modulation of glycemic responses (6, 7).

Building on this foundation, recent randomized controlled clinical trials have begun to incorporate microbiome profiling into personalized nutrition strategies, often leveraging machine -learning methods to predict individual PPGRs and generate tailored dietary recommendations. Zeevi et al. (6) integrated gut microbiome features with clinical, dietary, and lifestyle variables to develop an algorithm that accurately predicted PPGR to standardized and real life meals, and demonstrated that algorithm-based personalized diets could improve glycemic control compared with conventional dietary advice. The Personal Diet Study extended this precision nutrition framework to a weight loss setting by randomizing adults with prediabetes or moderately controlled T2D and overweight/obesity to one of two calorie restricted diets: a one-size-fits-all low-fat diet (*Standardized*) or a personalized diet guided by the PPGR prediction algorithm (*Personalized*) (8, 9). At 6 months, weight loss did not differ significantly between the two groups, underscoring the need to better understand the underlying biological and behavioral factors that may influence responsiveness to such interventions.

Within this context, the present *post-hoc*, cross-sectional analysis focuses on participants in the Personalized arm of the Personal Diet Study. Our aim was to comprehensively characterize the gut microbiome at baseline and to examine its associations with dietary intake, body composition, glycemic measures (continuous glucose monitoring–derived glycemic variability), and self-efficacy characteristics. By mapping gut microbial diversity and genus level profiles onto these multidimensional baseline factors in a high-risk metabolic population, we sought to identify key host and lifestyle correlates of gut microbiome structure that may inform future personalized nutrition and weight loss strategies.

## 2 Materials and methods

### 2.1 Study design

The data included in this *post-hoc* analysis were baseline data collected as part of The Personal Diet Study, a 6-month behavioral weight loss randomized controlled trial in adults with prediabetes and obesity (https://www.ClinicalTrials.gov, Identifier: NCT03336411). The study was conducted from January 2018 to March 2021 and was designed to compare the effects of *Personalized* and *Standardized* on body weight, body composition, resting energy expenditure (REE), respiratory quotient (RQ), metabolic adaptation, and glycemic variability (GV). For this analysis, data from only the *Personalized* arm were included, because stool samples were not collected in the *Standardized* arm. The trial was conducted in accordance with the Declaration of Helsinki (as revised in 2013) and was approved by the NYU Grossman School of Medicine Institutional Review Board (IRB #17-00741). Informed consent was obtained from all study participants. A detailed study design and intervention protocol have been published elsewhere (8) and are briefly described here.

### 2.2 Study sample

Participants in the Personal Diet Study were primarily recruited from the NYU Langone Health system via the MyChart patient portal. Eligible participants were 18 to 80 years of age, with overweight or obesity (body mass index [BMI]: 27 to 50 kg/m^2^) and prediabetes or moderately controlled T2D (hemoglobin A1c [HbA1c]: 5.7 to 8%) managed with lifestyle alone or lifestyle plus metformin. Considering the current study is cross-sectional, details on study intervention and randomization have been omitted and can be found elsewhere (9).

### 2.3 Sociodemographic and self-efficacy characteristics

Sociodemographic variables (age, sex, race, ethnicity, education, income), metformin use, and diagnosis of T2D were obtained by self-report at baseline. Weight loss self-efficacy was assessed at baseline using the validated 20-item Weight Efficacy Lifestyle (WEL) Questionnaire, which measures confidence in resisting eating across different situations (10). The WEL comprises 5 subscales—negative emotions, availability, social pressure, physical discomfort, and engaging in positive activities. Each item is rated on a 10-point visual numeric scale from 0 (not confident) to 9 (very confident). Total and subscale scores were calculated by summing the corresponding items.

### 2.4 Physical and metabolic characteristics and dietary intake

Body weight was measured either in person at NYU Langone Health’s Clinical and Translational Science Institute (CTSI) by trained research staff using a Scale-Tronix compact scale (Scale-Tronix Stow-A-Weight Scale; Welch Allyn) or, after the onset of the COVID-19 pandemic, remotely by participants using a Renpho Bluetooth-enabled smart scale (Renpho US). Body composition [fat mass (FM), body fat, and fat-free mass (FFM)] was assessed via bioelectrical impedance analysis (InBody 270 body composition analyzer; InBody, Inc.). REE and RQ were measured by open-circuit indirect calorimetry using a metabolic cart with a flow-dilution canopy hood (Quark RMR; COSMED). Details of these procedures have been described previously (8). Following pandemic-related protocol modifications, these outcomes were discontinued, so body composition and REE data were available only for a subset of participants. Because the Automated Self-Administered 24-Hour Dietary Assessment Tool (ASA24; 2018 and 2020 versions) (11) was implemented after study initiation, it was not included in the original assessment battery for all participants. Consequently, dietary intake was assessed in a subset of participants (*n* = 63).

### 2.5 Glycemic variability and HbA1c

HbA1c was initially obtained at NYU Langone Health’s Clinical and Translational Science Institute by venipuncture and analyzed using high performance liquid chromatography (Variant II Turbo analyzer). Following the onset of the COVID-19 pandemic, HbA1c values were obtained either from participants’ electronic medical records or using A1cNow point of care devices that were mailed to participants and used at home.

GV was assessed for up to 14 days using a blinded continuous glucose monitor (CGM; Abbott Freestyle Libre Pro, Abbott Park, IL, USA). In accordance with manufacturer guidance, the first 12 h of data after sensor insertion were excluded to avoid potential calibration-related inaccuracies. Any 24-h period in which the CGM failed to record ≥2 consecutive hours of glucose values was excluded because such gaps can yield unreliable mean amplitude of glycemic excursions (MAGE) estimates. After interpolating missing glucose values, GV metrics were calculated for each valid 24-h period using EasyGV version 9.0. R2 (Nathan R. Hill, University of Oxford, United Kingdom), and the mean across all CGM days was used for analysis. The GV measures included mean CGM glucose, SD (standard deviation), coefficient of variation (CV), MAGE, continuous overall net glycemic action (CONGA), hours per day with glucose above 140 mg/dL, and hours per day with glucose between 70 and 180 mg/dL (12). Because of pandemic-related protocol modifications, these outcomes were available only for a subset of participants (*n* = 75).

### 2.6 Fecal sample collection and microbiome assessment

At the baseline visit, participants were provided with an Omnigene stool collection kit (DNA Genotek, Inc., Ottawa, ON, Canada) used for gut microbiota profiling. Stool samples were collected during the pre-intervention visit and shipped to the Weizmann Institute (Rehovot, Israel) for microbiome analysis. Detailed stool collection procedures and sequencing/analytic methods are described elsewhere (6) and are briefly summarized here. Genomic DNA was purified using the PowerMag Soil DNA Isolation Kit (MoBio), optimized for the Tecan automated platform. Purified DNA (100 ng) was fragmented with a Covaris E220X sonicator, and Illumina-compatible libraries were prepared as previously described (13). Metagenomic reads containing Illumina adapter sequences were removed, low-quality reads were filtered out, and low-quality bases at read ends were trimmed. Host-derived DNA was identified by aligning reads to the human genome using GEM (14) with inclusive parameters, and matching reads were discarded. Taxonomic profiling and relative abundance estimation were performed on metagenomic sequencing data using MetaPhlAn2 (15) with default settings. Only samples with more than 10 million metagenomic reads were included in the analysis.

### 2.7 Statistical analysis

Baseline characteristics were summarized as mean (SD) for continuous variables and as counts (percentages) for categorical variables. Gut microbial community diversity was characterized using alpha diversity (Shannon index and observed richness) computed with the phyloseq package (16) and beta diversity (Jaccard index and Bray–Curtis dissimilarity) computed with the vegan package (17).

To assess associations between host/dietary factors and alpha diversity, we first fit separate simple linear regression models for each baseline predictor and each alpha diversity metric. Predictors with marginal evidence of association (*p* ≤ 0.20) in these univariable models were then entered into least absolute shrinkage and selection operator (LASSO) regression to perform data-driven variable selection for each alpha diversity outcome. All predictors were standardized before model fitting. The tuning parameter was selected by 10-fold cross-validation based on the minimum cross-validated error. We also performed 100 bootstrap resamples to improve the stability of variable selection. The five strongest predictors were then included in multivariable linear regression models fit to the original dataset to obtain interpretable regression coefficients and standard errors; standardized regression coefficients and *p*-values from these models were reported. Sensitivity analyses were also performed using covariate-adjusted models that additionally included age, sex, BMI, metformin use, and diabetes status, regardless of whether these variables were selected by LASSO.

Associations between host and dietary factors and beta diversity distance matrices were evaluated using univariate permutational multivariate analysis of variance (PERMANOVA). For categorical factors, the homogeneity of dispersion was assessed using the betadisper function. The proportion of variance explained (*R*^2^) and corresponding *p*-values were estimated using 10,000 permutations. Adjusted *p-*values were reported using Benjamini–Hochberg (BH) procedure to correct for multiple comparisons across all factors.

To explore relationships between the gut microbiota and host characteristics, we computed Spearman rank correlations between t genus-level microbial abundance and baseline clinical, dietary, anthropometric, emotional, and glycemic variables. Correlations were evaluated using both relative abundance and centered log-ratio (CLR)-transformed abundance. For each genus–variable pair, we obtained the correlation coefficient (*ρ*) and corresponding *p-*value. Adjusted *p-*values were obtained using BH procedure to correct for multiple comparisons across all genera. Genera and variables were retained for visualization only if only if they were involved in at least one statistically significant or trend-level association. The resulting correlations were displayed as a heatmap, with genera and baseline variables ordered by hierarchical clustering of the correlation matrix using the Ward.D2 linkage method (18).

We further quantified the gut microbial *Prevotella-to-Bacteroides* (*P/B*) ratio and assessed its relationships with diet. The P/B ratio was calculated as the log-transformed ratio of *Prevotella* to *Bacteroides* relative abundance, log(*Prevotella* relative abundance / *Bacteroides* relative abundance). Linear regression was used to examine the association between total energy intake and the *P/B* ratio, adjusting for age, sex, BMI, metformin use, and diabetes status. We also evaluated associations between the *P/B* ratio and individual dietary components using two dietary parameterizations: grams per day and percent of energy intake. Models for dietary components were additionally adjusted for total energy intake, along with age, sex, BMI, metformin use, and diabetes status. In all analyses, A result was considered statistically significant if its two-sided *p*-value or adjusted *p*-value <0.05 and indicative of a statistical trend if its two-sided *p*-value or adjusted *p-*value was ≥0.05 and <0.1. All statistical analyses were performed using R (R Core Team, R Foundation of Statistical Computing) version 4.4.3.

## 3 Results

### 3.1 Characteristics of participants in the Personalized arm

In the parent study, 103 participants were randomized to the Personalized arm (1 participant dropped out), with 95 of those participants having completed fecal sample collection. As shown in Supplementary Table 1, participant characteristics were generally similar between those with and without microbiome data, with no major systematic differences observed. Table 1 describes the characteristics of the 95 participants included in the present analysis. The mean age was 58 years (SD = 11.1), and most were female (75.8%), White (53.7%), and non-Hispanic (78.9%) with a mean BMI 34.6 kg/m^2^ (SD = 5.0) and a mean HbA1c of 5.7% (SD = 0.55). Reported energy intake averaged 1930 kcal/day from fat (36.9%, 80.3 g/day), carbohydrate (43.9%, 211 g/day), and protein (19.1%, 89.8 g/day).

**Table 1:** Characteristics of Personalized arm participants.

Category	Variable	Overall (*n* = 95)
Demographics	Age (y)	57.9 (11.1)
	Sex	
	Male	23 (24.2%)
	Female	72 (75.8%)
	Race (*n* = 93)	
	White	51 (53.7%)
	African American	20 (21.1%)
	Other	22 (23.2%)
	Ethnicity	
	Non-Hispanic	75 (78.9%)
	Hispanic	20 (21.1%)
Socioeconomics	Education (*n* = 89)	
	< Bachelor’s degree	27 (28.4%)
	≥ Bachelor’s degree	62 (65.3%)
	Employed	
	No	21 (22.1%)
	Yes	74 (77.9%)
	Income ($) (*n* = 81)	
	<75,000	29 (30.5%)
	>75,000	52 (54.7%)
Physical and metabolic characteristics	BMI (kg/m^2^)	34.6 (5.0)
	Body weight (kg)	94.7 (17.7)
	Fat free mass (kg) (*n* = 75)	55.1 (12.3)
	Fat mass (kg) (*n* = 75)	39.7 (10.3)
	Body fat percentage (%) (*n* = 75)	41.9 (7.77)
	Neck circumference (cm)	38.4 (4.17)
	Waist circumference (cm)	108 (11.5)
	Hip circumference (cm) (*n* = 94)	118 (10.0)
	Resting energy expenditure (*n* = 75)	1750 (372)
	Respiratory quotient (*n* = 75)	0.838 (0.121)
Self-efficacy	Negative emotion (*n* = 94)	22.3 (8.69)
	Availability (*n* = 93)	19.7 (7.92)
	Social pressure (*n* = 93)	25.0 (7.64)
	Physical discomfort (*n* = 93)	25.9 (7.02)
	Positive activities (*n* = 93)	26.6 (6.56)
	Total well-being score	118 (33.1)
Medication and T2D	Metformin use	
	No	77 (81.1%)
	Yes	18 (18.9%)
	T2D	
	No	76 (80.0%)
	Yes	19 (20.0%)
Glycemic variability	HbA1c	5.77 (0.55)
	MAGE (mg/dL) (*n* = 75)	51.2 (19.2)
	CONGA (mg/dL) (*n* = 75)	90.4 (17.3)
	Glucose: CV (mg/dL) (*n* = 75)	18.1 (4.97)
	Glucose: SD (mg/dL) (*n* = 75)	18.7 (6.96)
	Glucose: mean (mg/dL) (*n* = 75)	102 (18.9)
	Time >140 mg/dL (h/day) (*n* = 75)	8.04 (14.3)
	Time 70–180 mg/dL (h/day) (*n* = 75)	22.1 (3.24)
Dietary intake (*n* = 63)	Total energy intake (kilocalorie/day)	1930 (836)
	Total fat (gram/day)	80.3 (41.5)
	Total fat % energy	36.9 (9.04)
	Saturated fat (gram/day)	25.3 (16.0)
	Saturated fat % energy	11.5 (4.08)
	Monounsaturated fat (gram/day)	28.8 (16.4)
	Monounsaturated fat % energy	13.1 (4.62)
	Polyunsaturated fat (gram/day)	19.2 (11.7)
	Polyunsaturated fat % energy	9.02 (4.41)
	Carbohydrates (gram/day)	211 (108)
	Carbohydrates % energy	43.9 (11.0)
	Fiber (gram/day)	18.4 (11.3)
	Fiber % energy	2.02 (1.33)
	Sugar (gram/day)	90.3 (63.8)
	Sugar % energy	18.5 (9.56)
	Protein (gram/day)	89.8 (42.9)
	Protein % energy	19.1 (7.20)

For categories or variables with missing values, the number of participants with available data is reported.

### 3.2 Microbiome overall diversity

**Figure 1:**
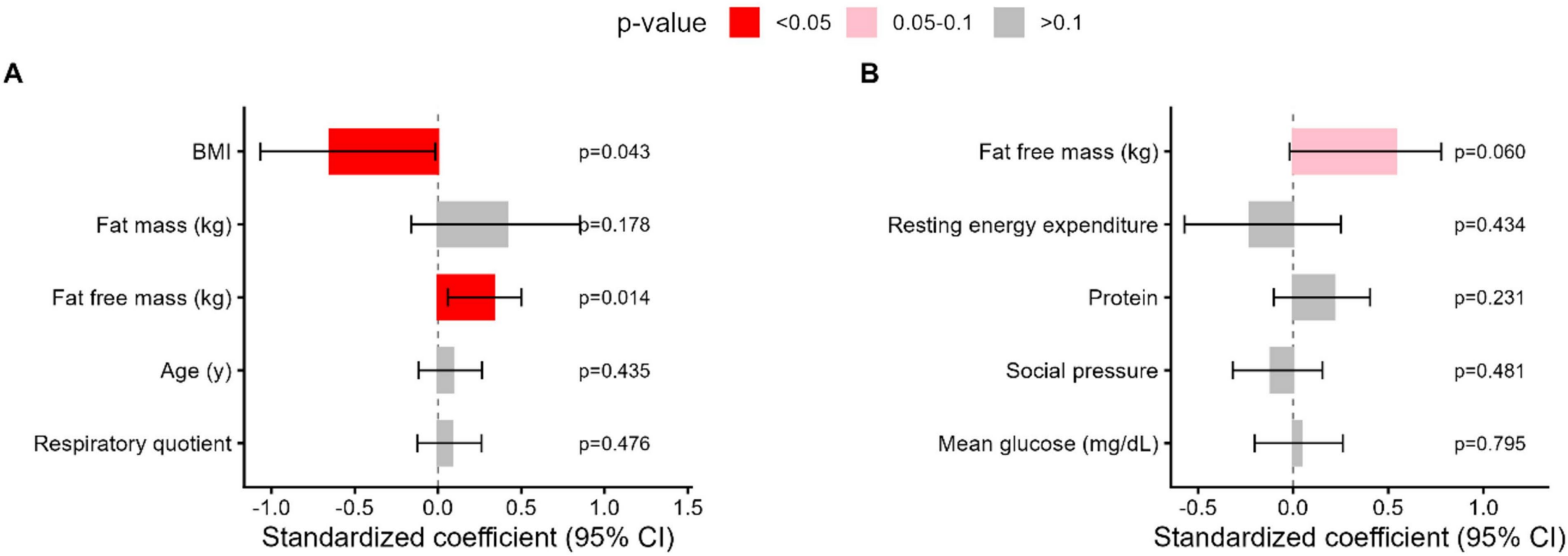
Associations between host and behavioral factors and gut microbial alpha diversity. Panels show standardized regression coefficients and 95% confidence intervals (*x*-axis) for the top five predictors selected for **(A)** Shannon diversity and **(B)** observed richness. Coefficients and *p-*values were estimated from multivariable linear regression models including the five strongest predictors identified by LASSO. All coefficients are standardized to allow comparison of effect magnitudes across predictors. Bars are color-coded by *p*-value (red: *p* < 0.05; pink: 0.05–0.10; grey: > 0.10). Among 95 participants, dietary intake was available for 63 participants, and metabolic characteristics and glycemic variability measures were available for 75 participants.

For Shannon diversity (Figure 1 and Supplementary Figure 1), higher FFM was significantly associated with greater diversity, whereas higher BMI was significantly associated with lower diversity (both *p* < 0.05). For observed richness, FFM showed a positive trend, although none of the selected variables reached statistical significance.

**Figure 2:**
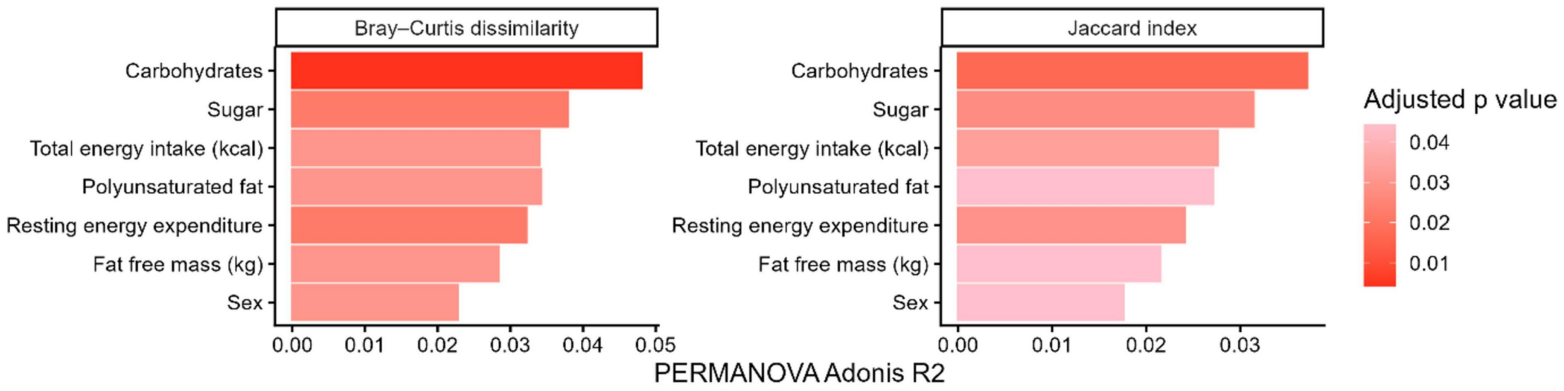
Univariate associations between host and dietary factors and gut microbial beta diversity. PERMANOVA was used to assess the proportion of variance in community structure explained by individual variables for Bray–Curtis dissimilarity (abundance-weighted) and the Jaccard index (presence–absence). Bars show the Adonis *R*^2^ values (*x*-axis) for each factor, with color intensity indicating statistical significance (all BH-adjusted *p* < 0.05, darker red corresponding to lower *p*-values). Among 95 participants, dietary intake was available for 63 participants, and metabolic characteristics and glycemic variability measures were available for 75 participants.

Figure 2 shows the variables significantly associated with gut microbial beta diversity for both Bray–Curtis dissimilarity and the Jaccard index (detailed numerical results for all factors are provided in Supplementary Table 2). Although effect sizes were modest, the significant variables were consistent across both metrics (adjusted *p*-values <0.05), with multiple dietary factors associated with community structure. Carbohydrates showed the largest proportion of explained variance, followed by sugar, total energy intake, polyunsaturated fat, REE, FFM, and sex.

### 3.3 Gut microbiota genera

**Figure 3:**
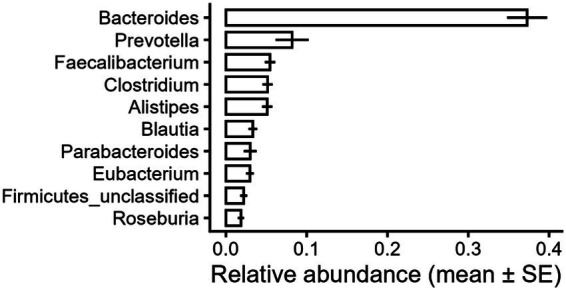
Mean relative abundance [± standard error (SE)] of the 10 most abundant bacterial genera in the study cohort.

A total of 302 bacterial genera were identified at baseline. To focus on prevalent and abundant taxa, we retained only genera with a mean relative abundance >0.001 and presence in >10% of samples and assigned taxonomic names; all others were excluded. After this filtering, 51 genera remained. Across participants, the 10 most abundant genera were *Bacteroides*, *Prevotella, Faecalibacterium, Clostridium, Alistipes, Blautia, Parabacteroides, Eubacterium, an unclassified Firmicutes genus,* and *Roseburia* (Figure 3 and Supplementary Figure 2). *Bacteroides* accounted for the largest fraction of sequences in most individuals, with *Prevotella* and *Faecalibacterium* typically comprising the next highest proportions. The remaining genera contributed to smaller, but consistent, portions of the community. Although the relative contribution of each genus varied across subjects, these 10 taxa together represented the majority of the gut bacterial community in this cohort.

**Figure 4:**
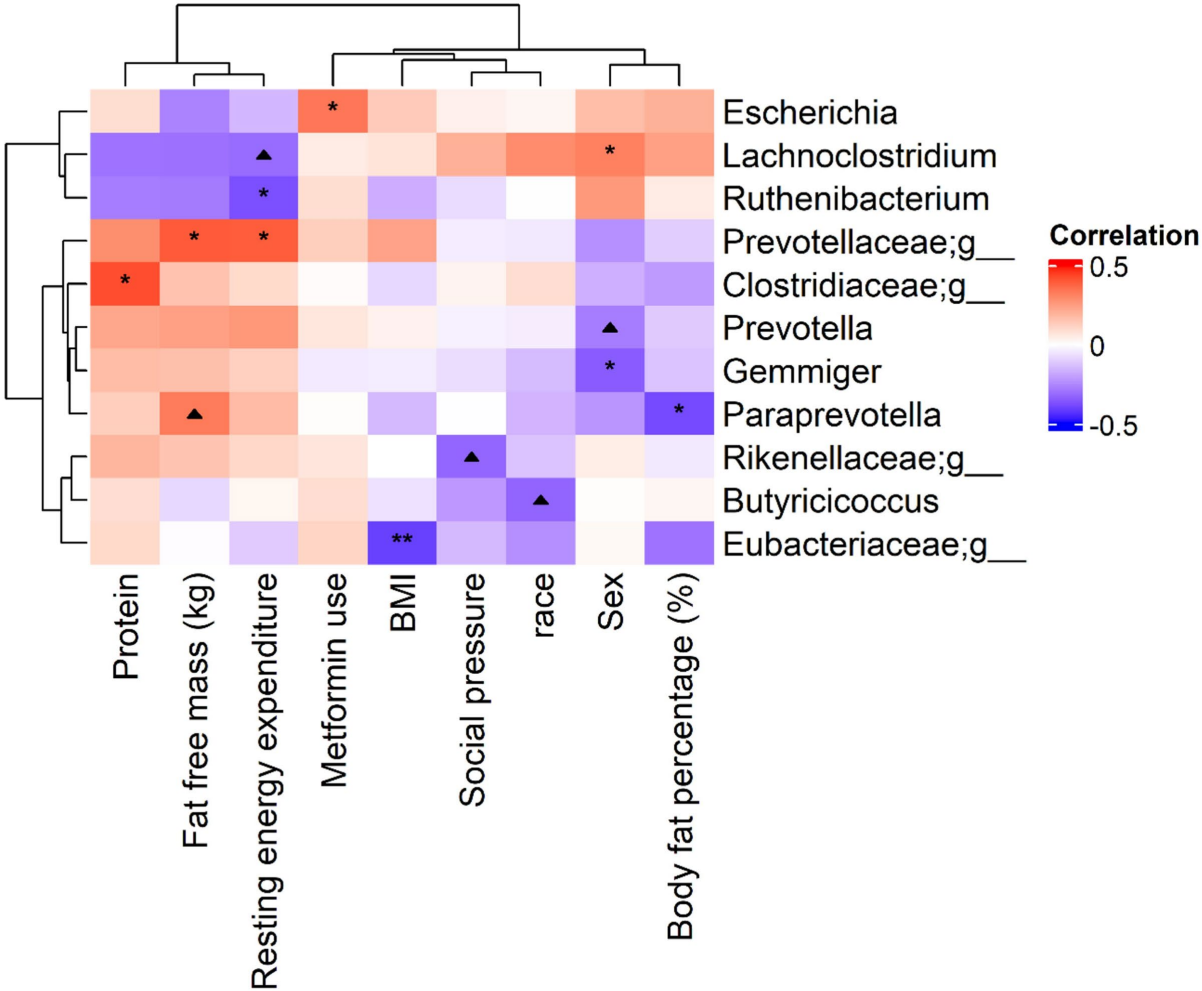
Spearman correlations between gut bacterial genera and baseline host and lifestyle characteristics. The heatmap displays Spearman rank correlation coefficients (*ρ*) between the relative abundance of each genus (rows) and baseline variables (columns). Adjusted *p-*values were calculated using Benjamini–Hochberg procedure across genera for each factor. Only genera and variables involved in at least one association with adjusted *p* < 0.1 are shown. Genera and variables are ordered by hierarchical clustering of the correlation matrix using the Ward. D2 method. Cell colors indicate the strength and direction of the correlation (red = positive, blue = negative). Triangles denote adjusted *p-*values between 0.05 and 0.10 (statistical trend), * indicates adjusted *p* < 0.05, and ** indicates adjusted *p* < 0.01. Among 95 participants, dietary intake was available for 63 participants, and metabolic characteristics and glycemic variability measures were available for 75 participants.

The correlation heatmap shows that several bacterial genera are associated with baseline host and lifestyle variables based on both relative abundance (RA) (Figure 4) and CLR-transformed abundance (Supplementary Figure 3). Notably, several associations are consistent across the two analytical approaches. Female sex is associated with higher *Lachnoclostridium* and lower *Gemmiger* (both adjusted *p-*values<0.05). Baseline metformin use is positively associated with *Escherichia* in the RA analysis and shows a similar trend in the CLR-based analysis. BMI is negatively associated with an unclassified genus in the family *Eubacteriaceae* (both adjusted *p*-values<0.01). Protein intake is positively associated with an unclassified genus in the family *Clostridiaceae* based on the RA analysis and shows a similar trend in the CLR-based analysis. In addition, resting energy expenditure is negatively associated with *Ruthenibacterium* across both analyses (adjusted *p*-values< 0.05 or 0.01).

Several additional associations were identified in only one of the two analytic approaches (adjusted *p*-values< 0.05), which may still be of interest given the modest sample size of this cohort. FFM is positively associated with an unclassified genus in the family *Prevotellaceae* in the RA analysis, whereas it was negatively associated with *Ruthenibacterium* in the CLR-based analysis. A positive association between an unclassified genus in the family *Prevotellaceae* and REE, as well as a negative association between body fat percentage and *Paraprevotella*, are observed only in the RA analysis. Neither glycemic measures nor self-efficacy was identified as being associated with any genera.

**Figure 5:**
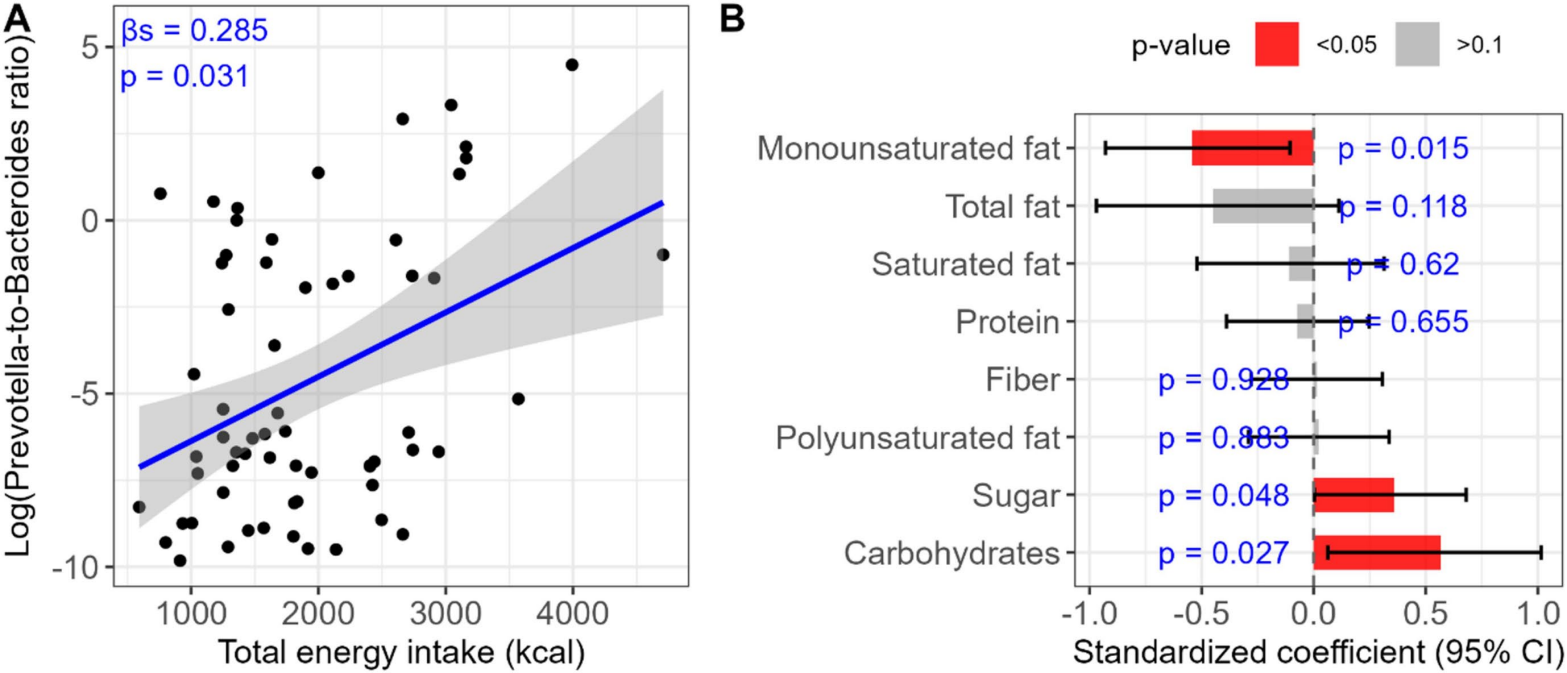
Associations between dietary intake and the *Prevotella*-to-*Bacteroides* (*P/B*) ratio. **(A)** Association between total energy intake (kcal) and the log-transformed *P/B* ratio. The blue line indicates the fitted linear regression, and the gray band represents the 95% confidence interval. The standardized regression coefficient and corresponding *p-*value are shown from a model adjusted for age, sex, BMI, metformin use, and diabetes status. **(B)** Associations between individual dietary components (g/day) and the P/B ratio from covariate-adjusted linear regression models. Bars show standardized regression coefficients and 95% confidence intervals. Models were adjusted for total energy intake, age, sex, BMI, metformin use, and diabetes status. Red bars indicate *p* < 0.05 and gray bars indicate *p* > 0.10. Among 95 participants, dietary intake was available for 63 participants, and metabolic characteristics and glycemic variability measures were available for 75 participants.

The *P/B* ratio is positively associated with total energy intake (standardized coefficient = 0.285, 95% CI: 0.026–0.516; *p* = 0.031; Figure 5A), after adjusting for age, sex, BMI, metformin use, and diabetes status. In addition, after adjusting for total energy intake, along with age, sex, BMI, metformin use, and diabetes status, the *P/B* ratio is positively associated with sugar intake (g/day) (standardized coefficient = 0.359, 95% CI: 0.003–0.681; *p* = 0.048) and carbohydrates intake (g/day) (standardized coefficient = 0.566, 95% CI: 0.063–1.015; *p* = 0.027), and negatively associated with monounsaturated fat (g/day) (standardized coefficient = −0.544, 95% CI: −0.929--0.106; *p* = 0.015) (Figure 5B). By contrast, none of the dietary components expressed as percentage of energy intake were significantly associated with the *P/B* ratio, although monounsaturated fat (%) showed a negative trend (Supplementary Figure 4).

## 4 Discussion

This *post-hoc*, cross-sectional analysis of the Personal Diet Study provides an integrated view of how reported dietary intake, body composition, metabolic status, glycemic control, sociodemographic factors, and self-efficacy characteristics are associated with the gut microbiome in adults with prediabetes or moderately controlled T2D and overweight or obesity. Higher FFM and lower BMI were associated with greater gut microbial alpha diversity, while beta diversity was mainly associated with diet. The microbiome was dominated by *Bacteroides*, *Prevotella*, and *Faecalibacterium*, and the *P/B* ratio was positively associated with total energy intake, sugar and carbohydrate intake, while negatively associated monounsaturated fat intake. Overall, different genera exhibit distinct correlation signatures across clusters of baseline variables, with the most consistent associations observed for dietary intake and measures of body composition and metabolic status, whereas glycemic measures and self-efficacy were not associated with any genera in this analysis.

These findings support prior evidence linking lower diversity to obesity and T2D, reinforce diet as a primary determinant of microbiome composition, and highlight body composition and energy metabolism as important correlates of microbial structure (19–21). Prior studies suggest a link between alpha-diversity and lean body mass in humans. Barger et al. (22) reported that butyrate-producing bacteria and genes were associated with greater skeletal muscle mass. Mechanistically, the production of butyrate, a short chain fatty acid, may improve muscle health through lower inflammation, improved insulin sensitivity, and better physical performance. Some studies imply that better-preserved lean body mass correlates with microbial diversity metrics, potentially via physical activity or diet-mediated pathways that may also influence FFM (23–25).

In contrast, our findings indicate an inverse relationship between BMI and alpha diversity, a relationship that is most likely influenced by multiple interacting factors, such as diet quality, fiber intake, and physical activity. Sun et al. (26) examined the gut microbiome of vegetarians and omnivores, and found higher alpha diversity in vegetarians who had lower BMIs; thus, indicating a diet and BMI-linked pattern in alpha diversity. The association of BMI and alpha diversity appears more variable but tends toward an inverse relationship in many cohorts, with obesity more frequently linked to reduced alpha diversity.

In line with prior studies, our findings show that diet has more strong associations with gut microbiota beta diversity, than other host or lifestyle factors. Specific dietary components consistently contribute to explaining variance in adult gut microbiota beta diversity. As with prior findings (27–29), we found that carbohydrates explained the greatest proportion of variance for gut microbial diversity (both Bray–Curtis dissimilarity and Jaccard index). Sugar, total energy intake, and polyunsaturated fat were also significantly associated with community structure, along with REE, FFM, and sex. Although effect sizes were modest, the consistency across both beta-diversity metrics strengthens confidence in these associations.

Our results also support the notion that dietary patterns may shift the gut microbiome along a *Prevotella*-*Bacteroides* continuum. In our analysis, a higher *P/B* ratio was associated with greater total energy intake and, after adjustment for total energy intake, with higher sugar and carbohydrate intake and lower monounsaturated fat intake. By contrast, dietary components expressed as percentage of energy intake were not significantly associated with the ratio, although monounsaturated fat percentage showed a negative trend. These findings are broadly consistent with prior work suggesting that the relative balance of *Prevotella* and *Bacteroides* may reflect dietary patterning (29–32), but our data do not establish stable enterotype-like groupings or causal dietary effects.

Future work should leverage longitudinal data from similar trials to determine whether baseline microbiome features predict response to personalized diet interventions and how the microbiome changes with weight loss, diet modification, and shifts in body composition. Integrating metagenomic and metabolomic profiling with continuous glucose monitoring and detailed dietary assessment could clarify mechanisms linking diet, microbiome function, and glycemic control, and help identify microbiome-based targets for intervention. Larger and more diverse cohorts will be essential to test generalizability and to explore whether specific microbiome–diet–glycemia patterns differ across populations.

This study has limitations. First, the cross-sectional design precludes causal inference. Second, the modest sample size limits power to detect smaller effects and interactions. Third, this *post-hoc* analysis was restricted to participants in the Personalized arm from a single health system, which may limit generalizability. In addition, several variables, including body composition, metabolic/glycemic measures, and dietary intake, were available only in subsets of participants. Consequently, the analytic sample varied across models, which may have introduced bias; findings for variables with greater missingness should therefore be interpreted cautiously. Finally, dietary intake was assessed using a single 24-h recall in a subset of participants and may not reflect habitual intake, given day-to-day variability and potential reporting error. Accordingly, the observed diet–microbiome associations should be interpreted as cross-sectional correlates rather than evidence of stable dietary effects. Despite these limitations, this study provides an integrated view of how diet, body composition, glycemic dynamics, and self-efficacy characteristics relate to the gut microbiome in a clinically relevant high-risk population. These results support further investigation of microbiome-informed personalized nutrition approaches, while emphasizing the need for longitudinal and methodologically robust studies to establish the direction, stability, and clinical relevance of these associations.

## 5 Conclusion

In this *post-hoc* cross-sectional analysis of adults with prediabetes and obesity, baseline gut microbiome variation was most strongly related to dietary intake, with additional associations observed for body composition and selected host characteristics. Dietary factors were the principal correlates of beta diversity, while higher fat-free mass and lower BMI were associated with greater Shannon diversity. Genus-level associations were observed for sex, metformin use, BMI, protein intake, and resting energy expenditure, whereas glycemic measures and self-efficacy were not associated with any genera in this cohort. The *Prevotella/Bacteroides* ratio was also associated with total energy, sugar, carbohydrate, and monounsaturated fat intake. Together, these findings underscore diet as a key correlate of gut microbiome structure in this metabolically vulnerable population and motivate further research on microbiome-informed precision nutrition approaches for obesity prevention and management.

## Data Availability

The raw data supporting the conclusions of this article will be made available by the authors, without undue reservation.

## References

[1] Vishwakarma RK Gautam P Sahu M Nath G Yadav BS. Gut microbiome in obesity: a narrative review of mechanisms, interventions, and future directions. Probiotics Antimicrob Proteins. (2025) 18:5223–45. doi: 10.1007/s12602-025-10855-1, 41313537

[2] Castaner O Goday A Park Y-M Lee S-H Magkos F Shiow S-ATE . The gut microbiome profile in obesity: a systematic review. Int J Endocrinol. (2018) 2018:1–9. doi: 10.1155/2018/4095789, 29849617 PMC5933040

[3] Delzenne NM Bindels LB Neyrinck AM Walter J. The gut microbiome and dietary fibres: implications in obesity, cardiometabolic diseases and cancer. Nat Rev Microbiol. (2025) 23:225–38. doi: 10.1038/s41579-024-01108-z, 39390291

[4] Muijsenberg A Canfora EE Blaak EE. Metabolic phenotypes, genotypes, and gut microbiome signatures in obesity: implications for precision nutrition strategies in type 2 diabetes prevention. Nutr Rev. (2026) 84:158–88. doi: 10.1093/nutrit/nuaf088, 40587382 PMC12696375

[5] Ludwig DS Ebbeling CB. The carbohydrate-insulin model of obesity: beyond "calories in, calories out". JAMA Intern Med. (2018) 178:1098–103. doi: 10.1001/jamainternmed.2018.2933, 29971406 PMC6082688

[6] Zeevi D Korem T Zmora N Israeli D Rothschild D Weinberger A . Personalized nutrition by prediction of glycemic responses. Cell. (2015) 163:1079–94. doi: 10.1016/j.cell.2015.11.001, 26590418

[7] David LA Maurice CF Carmody RN Gootenberg DB Button JE Wolfe BE . Diet rapidly and reproducibly alters the human gut microbiome. Nature. (2014) 505:559–63. doi: 10.1038/nature12820, 24336217 PMC3957428

[8] Popp CJ St-Jules DE Hu L Ganguzza L Illiano P Curran M . The rationale and design of the personal diet study, a randomized clinical trial evaluating a personalized approach to weight loss in individuals with pre-diabetes and early-stage type 2 diabetes. Contemp Clin Trials. (2019) 79:80–8. doi: 10.1016/j.cct.2019.03.001, 30844471

[9] Popp CJ Hu L Kharmats AY Curran M Berube L Wang C . Effect of a personalized diet to reduce postprandial glycemic response vs a low-fat diet on weight loss in adults with abnormal glucose metabolism and obesity: a randomized clinical trial. JAMA Netw Open. (2022) 5:e2233760. doi: 10.1001/jamanetworkopen.2022.33760, 36169954 PMC9520362

[10] Clark MM Abrams DB Niaura RS Eaton CA Rossi JS. Self-efficacy in weight management. J Consult Clin Psychol. (1991) 59:739–44. doi: 10.1037/0022-006X.59.5.739, 1955608

[11] Subar AF Kirkpatrick SI Mittl B Zimmerman TP Thompson FE Bingley C . The automated self-administered 24-hour dietary recall (ASA24): a resource for researchers, clinicians, and educators from the National Cancer Institute. J Acad Nutr Diet. (2012) 112:1134–7. doi: 10.1016/j.jand.2012.04.016, 22704899 PMC3721511

[12] Battelino T Danne T Bergenstal RM Amiel SA Beck R Biester T . Clinical targets for continuous glucose monitoring data interpretation: recommendations from the international consensus on time in range. Diabetes Care. (2019) 42:1593–603. doi: 10.2337/dci19-0028, 31177185 PMC6973648

[13] Suez J Korem T Zeevi D Zilberman-Schapira G Thaiss CA Maza O . Artificial sweeteners induce glucose intolerance by altering the gut microbiota. Nature. (2014) 514:181–6. doi: 10.1038/nature13793, 25231862

[14] Marco-Sola S Sammeth M Guigó R Ribeca P. The GEM mapper: fast, accurate and versatile alignment by filtration. Nat Methods. (2012) 9:1185–8. doi: 10.1038/nmeth.2221, 23103880

[15] Truong DT Franzosa EA Tickle TL Scholz M Weingart G Pasolli E . MetaPhlAn2 for enhanced metagenomic taxonomic profiling. Nat Methods. (2015) 12:902–3. doi: 10.1038/nmeth.3589, 26418763

[16] McMurdie PJ Holmes S. Phyloseq: an R package for reproducible interactive analysis and graphics of microbiome census data. PLoS One. (2013) 8:e61217. doi: 10.1371/journal.pone.0061217, 23630581 PMC3632530

[17] Dixon P. VEGAN, a package of R functions for community ecology. J Veg Sci. (2003) 14:927–30. doi: 10.1111/j.1654-1103.2003.tb02228.x

[18] Murtagh F Contreras P. Algorithms for hierarchical clustering: an overview. WIREs Data Min Knowl Discov. (2012) 2:86–97. doi: 10.1002/widm.53

[19] Aydin Ö Nieuwdorp M Gerdes V. The gut microbiome as a target for the treatment of type 2 diabetes. Curr Diab Rep. (2018) 18:55. doi: 10.1007/s11892-018-1020-6, 29931613 PMC6013535

[20] Clemente-Suarez VJ . New insights and potential therapeutic interventions in metabolic diseases. Int J Mol Sci. (2023) 24:10672. doi: 10.3390/ijms241310672, 37445852 PMC10342188

[21] Enache RM Profir M Roşu OA Creţoiu SM Gaspar BS. The role of gut microbiota in the onset and progression of obesity and associated comorbidities. Int J Mol Sci. (2024) 25:12321. doi: 10.3390/ijms252212321, 39596385 PMC11595101

[22] Barger K Langsetmo L Orwoll ES Lustgarten MS. Investigation of the diet-gut-muscle axis in the osteoporotic fractures in men study. J Nutr Health Aging. (2020) 24:445–52. doi: 10.1007/s12603-020-1344-1, 32242213 PMC7524010

[23] Knoll RL Jarquín-Díaz VH Klopp J Kemper A Hilbert K Hillen B . Resilience and stability of the CF-intestinal and respiratory microbiome during nutritional and exercise intervention. BMC Microbiol. (2023) 23:44. doi: 10.1186/s12866-023-02788-y, 36803565 PMC9942320

[24] Boisseau N Barnich N Koechlin-Ramonatxo C. The nutrition-microbiota-physical activity triad: an inspiring new concept for health and sports performance. Nutrients. (2022) 14:924. doi: 10.3390/nu14050924, 35267899 PMC8912693

[25] Ones E . Effects of kefir consumption on gut microbiota and athletic performance in professional female soccer players: a randomized controlled trial. Nutrients. (2025) 17:512. doi: 10.3390/nu17030512, 39940370 PMC11820909

[26] Sun C Li A Xu C Ma J Wang H Jiang Z . Comparative analysis of fecal microbiota in vegetarians and omnivores. Nutrients. (2023) 15:2358. doi: 10.3390/nu15102358, 37242241 PMC10221195

[27] Cotillard A Cartier-Meheust A Litwin NS Chaumont S Saccareau M Lejzerowicz F . A posteriori dietary patterns better explain variations of the gut microbiome than individual markers in the American gut project. Am J Clin Nutr. (2022) 115:432–43. doi: 10.1093/ajcn/nqab332, 34617562 PMC8827078

[28] Troci A Rausch P Waschina S Lieb W Franke A Bang C. Long-term dietary effects on human gut microbiota composition employing shotgun metagenomics data analysis. Mol Nutr Food Res. (2023) 67:e2101098. doi: 10.1002/mnfr.202101098, 35760036

[29] Gou W Miao Z Deng K Zheng JS. Nutri-microbiome epidemiology, an emerging field to disentangle the interplay between nutrition and microbiome for human health. Protein Cell. (2023) 14:787–806. doi: 10.1093/procel/pwad023, 37099800 PMC10636640

[30] Arora T Backhed F. The gut microbiota and metabolic disease: current understanding and future perspectives. J Intern Med. (2016) 280:339–49. doi: 10.1111/joim.12508, 27071815

[31] Jardon KM Canfora EE Goossens GH Blaak EE. Dietary macronutrients and the gut microbiome: a precision nutrition approach to improve cardiometabolic health. Gut. (2022) 71:1214–26. doi: 10.1136/gutjnl-2020-323715, 35135841 PMC9120404

[32] Garcia-Vega AS . Diet quality, food groups and nutrients associated with the gut microbiota in a nonwestern population. Nutrients. (2020) 12:2938. doi: 10.3390/nu12102938, 32992776 PMC7600083

