# Supplementary material for: Associations between the gut microbiome and diet, body composition, and glycemic profiles: a cross-sectional post-hoc analysis of the Personal Diet Study
Source: Front Nutr. 2026 Aug 3;13:1851439. doi: 10.3389/fnut.2026.1851439 (PMC13478278; doi:10.3389/fnut.2026.1851439)
Supplement: Supplementary file 1 [file Data_Sheet_1.DOCX]

**Supplementary Table 1. Participant characteristics in the Personalized arm with and without available microbiome data**

|  |  | Participants with microbiome (n=95) | Participants without microbiome (n=8) | P-value |
| --- | --- | --- | --- | --- |
| Demographics | Age (y) | 57.9 (11.1) | 55.9 (5.91) | 0.402 |
|  | Sex |  |  | 0.202 |
|  | Male | 23 (24.2%) | 4 (50.0%) |  |
|  | Female | 72 (75.8%) | 4 (50.0%) |  |
|  | Race |  |  | 0.467 |
|  | White | 51 (53.7%) | 3 (37.5%) |  |
|  | African American/Other | 42 (44.2%) | 5 (62.5%) |  |
|  | Ethnicity |  |  | 0.349 |
|  | Non-Hispanic | 75 (78.9%) | 8 (100%) |  |
|  | Hispanic | 20 (21.1%) | 0 (0%) |  |
| Socioeconomics | Education | |  | 1 |
|  | < Bachelor’s Degree | 27 (28.4%) | 2 (25.0%) |  |
|  | ≥ Bachelor’s Degree | 62 (65.3%) | 5 (62.5%) |  |
|  | Employment | |  | 1 |
|  | No | 21 (22.1%) | 2 (25.0%) |  |
|  | Yes | 74 (77.9%) | 6 (75.0%) |  |
|  | Income ($) | |  | 0.702 |
|  | <75,000 | 29 (30.5%) | 3 (37.5%) |  |
|  | >75,000 | 52 (54.7%) | 4 (50.0%) |  |
| Medication and T2D | Metformin use | |  | 1 |
|  | No | 77 (81.1%) | 7 (87.5%) |  |
|  | Yes | 18 (18.9%) | 1 (12.5%) |  |
|  | T2D |  |  | 0.363 |
|  | No | 76 (80.0%) | 5 (62.5%) |  |
|  | Yes | 19 (20.0%) | 3 (37.5%) |  |
| Physical and metabolic characteristics | BMI (kg/m2) | 34.6 (4.99) | 35.5 (4.35) | 0.583 |
|  | Body weight (kg) | 94.7 (17.7) | 105 (19.6) | 0.174 |
|  | Fat free mass (kg) | 55.1 (12.3) | 61.8 (13.6) | 0.249 |
|  | Fat mass (kg) | 39.7 (10.3) | 40.6 (10.0) | 0.831 |
|  | Body fat percentage (%) | 41.9 (7.77) | 39.7 (6.68) | 0.439 |
|  | Resting energy expenditure | 1750 (372) | 1830 (428) | 0.617 |
|  | Respiratory quotient | 0.838 (0.121) | 0.866 (0.0959) | 0.496 |
|  | Neck circumference (cm) | 38.4 (4.17) | 39.9 (4.17) | 0.366 |
|  | Waist circumference (cm) | 108 (11.5) | 114 (11.9) | 0.261 |
|  | Hip circumference (cm) | 118 (10.0) | 120 (9.03) | 0.6 |
| Glycemic variability | HbA1c | 5.77 (0.554) | 5.84 (0.350) | 0.613 |
|  | MAGE (mg/dL) | 51.2 (19.2) | 47.7 (11.5) | 0.523 |
|  | CONGA (mg/dL) | 90.4 (17.3) | 78.6 (10.4) | 0.0378 |
|  | Glucose: CV (mg/dL) | 18.1 (4.97) | 19.0 (5.46) | 0.695 |
|  | Glucose: SD (mg/dL) | 18.7 (6.96) | 17.4 (6.13) | 0.636 |
|  | Glucose: mean (mg/dL) | 102 (18.9) | 90.6 (12.1) | 0.0661 |
|  | Time >140 mg/dL (h/day) | 8.04 (14.3) | 2.75 (3.60) | 0.0254 |
|  | Time 70–180 mg/dL (h/day) | 22.1 (3.24) | 20.3 (5.43) | 0.468 |
| Dietary intake | Total energy intake (kilocalorie/day) | 1930 (836) | 2970 (1210) | 0.435 |
|  | Fiber (gram/day) | 18.4 (11.3) | 30.4 (26.4) | 0.637 |
|  | Saturated fat (gram/day) | 25.3 (16.0) | 36.4 (7.02) | 0.232 |
|  | Total fat (gram/day) | 80.3 (41.5) | 136 (44.2) | 0.321 |
|  | Monounsaturated fat (gram/day) | 28.8 (16.4) | 50.4 (17.0) | 0.316 |
|  | Polyunsaturated fat (gram/day) | 19.2 (11.7) | 36.0 (13.0) | 0.313 |
|  | Carbohydrates (gram/day) | 211 (108) | 298 (163) | 0.586 |
|  | Sugar (gram/day) | 90.3 (63.8) | 114 (69.2) | 0.708 |
|  | Protein (gram/day) | 89.8 (42.9) | 145 (51.6) | 0.365 |
| Self-efficacy | Negative emotion | 22.3 (8.69) | 23.5 (8.60) | 0.721 |
|  | Availability | 19.7 (7.92) | 21.6 (8.14) | 0.544 |
|  | Social pressure | 25.0 (7.64) | 26.9 (5.30) | 0.39 |
|  | Physical discomfort | 25.9 (7.02) | 26.1 (7.55) | 0.926 |
|  | Positive activities | 26.6 (6.56) | 26.0 (5.10) | 0.766 |
|  | Total well-being score | 118 (33.1) | 124 (29.0) | 0.583 |

Note: P-values were calculated by using t test for the continuous variables, and Fisher’s exact test for the categorical variables.

**Supplementary Table 2.** Univariable PERMANOVA results for associations of host and dietary factors with gut microbiome beta diversity (Bray–Curtis and Jaccard distances). Reported values include R², p-values, and BH-adjusted p-values based on 10,000 permutations. For categorical variables, betadisper p-values are also provided.

| **Variable** | **R2** | **P value** | **Adjusted P value** | **homogeneity of dispersion**  **p-value** |
| --- | --- | --- | --- | --- |
| **Bray-Curtis dissimilarity** | | |  |  |
| Carbohydrates | 0.048 | 1.00E-04 | 0.0042 | NA |
| Resting energy expenditure | 0.0322 | 0.0015 | 0.0224 | NA |
| Sugar | 0.0379 | 0.0016 | 0.0224 | NA |
| Total energy intake (kcal) | 0.034 | 0.0037 | 0.03 | NA |
| Fat free mass (kg) | 0.0284 | 0.0047 | 0.03 | NA |
| Sex | 0.0227 | 0.0048 | 0.03 | 0.5478 |
| Polyunsaturated fat | 0.0342 | 0.005 | 0.03 | NA |
| Fiber | 0.0263 | 0.031 | 0.1628 | NA |
| Neck circumference (cm) | 0.0167 | 0.0516 | 0.2408 | NA |
| Total fat | 0.0243 | 0.0594 | 0.243 | NA |
| Saturated fat | 0.0234 | 0.0695 | 0.243 | NA |
| employment | 0.0157 | 0.0723 | 0.243 | 0.3334 |
| race | 0.0158 | 0.0786 | 0.243 | 0.0378 |
| Body fat percentage (%) | 0.0191 | 0.081 | 0.243 | NA |
| Hip circumference (cm) | 0.0138 | 0.1491 | 0.4008 | NA |
| Body weight (kg) | 0.0136 | 0.1527 | 0.4008 | NA |
| Social pressure | 0.0138 | 0.1623 | 0.401 | NA |
| BMI | 0.0127 | 0.2118 | 0.4892 | NA |
| Protein | 0.0192 | 0.2213 | 0.4892 | NA |
| Monounsaturated fat | 0.0183 | 0.2785 | 0.5849 | NA |
| Waist circumference (cm) | 0.0118 | 0.299 | 0.598 | NA |
| Ethnicity | 0.0113 | 0.3412 | 0.6372 | 0.3721 |
| Positive activities | 0.0115 | 0.3572 | 0.6372 | NA |
| Fat mass (kg) | 0.0143 | 0.3641 | 0.6372 | NA |
| Education | 0.011 | 0.482 | 0.8098 | 0.8025 |
| Metformin use | 0.0099 | 0.5466 | 0.883 | 0.868 |
| Availability | 0.0096 | 0.6051 | 0.9413 | NA |
| Respiratory quotient | 0.0117 | 0.6483 | 0.9669 | NA |
| Age (y) | 0.0082 | 0.7745 | 0.9669 | NA |
| Income | 0.0094 | 0.7923 | 0.9669 | 0.8379 |
| hbA1c | 0.008 | 0.8075 | 0.9669 | NA |
| Time >140 mg/dL (h/day) | 0.01 | 0.8263 | 0.9669 | NA |
| Physical discomfort | 0.0077 | 0.8526 | 0.9669 | NA |
| Total well-being score | 0.0076 | 0.8553 | 0.9669 | NA |
| Negative emotion | 0.0075 | 0.8669 | 0.9669 | NA |
| T2D | 0.0072 | 0.8951 | 0.9669 | 0.8086 |
| Mean glucose (mg/dL) | 0.0091 | 0.8973 | 0.9669 | NA |
| Time 70-180 mg/dL (h/day) | 0.0087 | 0.931 | 0.9669 | NA |
| CONGA (mg/dL) | 0.0084 | 0.932 | 0.9669 | NA |
| Glucose SD (mg/dL) | 0.0086 | 0.9351 | 0.9669 | NA |
| MAGE (mg/dL) | 0.0081 | 0.9528 | 0.9669 | NA |
| Glucose CV (%) | 0.0079 | 0.9669 | 0.9669 | NA |
| **Jaccard index** | | | | |
| Carbohydrates | 0.0371 | 4.00E-04 | 0.0168 | NA |
| Sugar | 0.0314 | 0.0013 | 0.0273 | NA |
| Resting energy expenditure | 0.0241 | 0.0021 | 0.0294 | NA |
| Total energy intake (kcal) | 0.0276 | 0.0032 | 0.0336 | NA |
| Polyunsaturated fat | 0.0271 | 0.0053 | 0.0444 | NA |
| Sex | 0.0176 | 0.007 | 0.0444 | 0.3178 |
| Fat free mass (kg) | 0.0215 | 0.0074 | 0.0444 | NA |
| Fiber | 0.0222 | 0.0388 | 0.2037 | NA |
| Total fat | 0.0216 | 0.0474 | 0.2212 | NA |
| employment | 0.014 | 0.0605 | 0.2356 | 0.2585 |
| Saturated fat | 0.0209 | 0.0617 | 0.2356 | NA |
| Neck circumference (cm) | 0.0138 | 0.0758 | 0.2653 | NA |
| race | 0.0135 | 0.1009 | 0.326 | 0.0455 |
| Body fat percentage (%) | 0.0162 | 0.1357 | 0.4071 | NA |
| Monounsaturated fat | 0.0183 | 0.1926 | 0.5137 | NA |
| Body weight (kg) | 0.0121 | 0.1957 | 0.5137 | NA |
| Social pressure | 0.0123 | 0.2118 | 0.5168 | NA |
| Hip circumference (cm) | 0.012 | 0.2215 | 0.5168 | NA |
| BMI | 0.0117 | 0.253 | 0.5593 | NA |
| Protein | 0.0174 | 0.2727 | 0.5727 | NA |
| Waist circumference (cm) | 0.0113 | 0.3118 | 0.6236 | NA |
| Fat mass (kg) | 0.014 | 0.3631 | 0.6649 | NA |
| Positive activities | 0.0112 | 0.3641 | 0.6649 | NA |
| Ethnicity | 0.0109 | 0.3815 | 0.6676 | 0.1978 |
| Education | 0.0112 | 0.4718 | 0.7926 | 0.5983 |
| Metformin use | 0.0101 | 0.5568 | 0.8994 | 0.8479 |
| Availability | 0.0101 | 0.6057 | 0.9282 | NA |
| Respiratory quotient | 0.0125 | 0.6188 | 0.9282 | NA |
| Age (y) | 0.0093 | 0.7277 | 0.955 | NA |
| Time >140 mg/dL (h/day) | 0.0116 | 0.7888 | 0.955 | NA |
| Income | 0.0104 | 0.8155 | 0.955 | 0.7414 |
| hbA1c | 0.0088 | 0.8395 | 0.955 | NA |
| Total well-being score | 0.0087 | 0.8524 | 0.955 | NA |
| Physical discomfort | 0.0087 | 0.859 | 0.955 | NA |
| Mean glucose (mg/dL) | 0.0108 | 0.8842 | 0.955 | NA |
| T2D | 0.0084 | 0.8986 | 0.955 | 0.5343 |
| Negative emotion | 0.0085 | 0.9032 | 0.955 | NA |
| Glucose SD (mg/dL) | 0.0106 | 0.9132 | 0.955 | NA |
| MAGE (mg/dL) | 0.0105 | 0.9225 | 0.955 | NA |
| CONGA (mg/dL) | 0.0104 | 0.9238 | 0.955 | NA |
| Time 70-180 mg/dL (h/day) | 0.0104 | 0.9375 | 0.955 | NA |
| Glucose CV (%) | 0.0101 | 0.955 | 0.955 | NA |

Note: All categorical variables satisfied the homogeneity of dispersion assumption except for sex; however, sex was not significantly associated with either Bray–Curtis or Jaccard distances in the PERMANOVA analysis.

**Supplementary Figure 1**. Sensitivity analysis of associations of host and behavioral factors with gut microbial alpha diversity after additional covariate adjustment. Panels show standardized regression coefficients and 95% confidence intervals (x-axis) from multivariable linear regression models for (A) Shannon diversity and (B) observed richness. Models included the top predictors identified by LASSO, with additional adjustment for age, sex, BMI, metformin use, and type 2 diabetes status regardless of LASSO selection. All coefficients were standardized to allow comparison of effect sizes across predictors. Bars are color‑coded by p‑value (red: p < 0.05; pink: 0.05–0.10; grey: > 0.10).

**Supplementary Figure 2**. Relative abundance of the top 10 bacterial genera in the gut microbiota across all participants.

**Supplementary Figure 3**. Sensitivity analysis of spearman correlations between gut bacterial genera and baseline host and lifestyle characteristics. The heatmap displays Spearman rank correlation coefficients (ρ) between the CLR-transformed relative abundance of each genus (rows) and baseline variables (columns). Adjusted p values were calculated using Benjamini–Hochberg false discovery rate (FDR) correction across genera for each factor. Only genera and variables involved in at least one association with adjusted p < 0.1 are shown. Genera and variables are ordered by hierarchical clustering of the correlation matrix using the Ward.D2 method. Cell colors indicate the strength and direction of the correlation (red = positive, blue = negative). Triangles denote adjusted p values between 0.05 and 0.10 (statistical trend), * indicates adjusted p <0.05, and ** indicates adjusted p < 0.01.

**Supplementary Figure 4**. Associations between individual dietary components (% of energy intake) and the *Prevotella*-to-*Bacteroides* ratio from covariate-adjusted linear regression models. Bars show standardized regression coefficients and 95% confidence intervals. Models were adjusted for total energy intake, age, sex, BMI, metformin use, and diabetes status. Pink bars indicate p values between 0.05 and 0.10 and gray bars indicate p > 0.10.
